# Full-thickness cartilage defects are repaired via a microfracture technique and intraarticular injection of the small-molecule compound kartogenin

**DOI:** 10.1186/s13075-015-0537-1

**Published:** 2015-02-02

**Authors:** Xingquan Xu, Dongquan Shi, Yeshuai Shen, Zhihong Xu, Jin Dai, Dongyang Chen, Huajian Teng, Qing Jiang

**Affiliations:** The Center of Diagnosis and Treatment for Joint Disease, Drum Tower Hospital, Medical School, Nanjing University, Zhongshan Road 321, Nanjing, 210008 Jiangsu China; Joint Research Center for Bone and Joint Disease, Model Animal Research Center (MARC), Nanjing University, Nanjing, 210093 Jiangsu China

## Abstract

**Introduction:**

Microfracture does not properly repair full-thickness cartilage defects. The purpose of this study was to evaluate the effect of intraarticular injection of the small-molecule compound kartogenin (KGN) on the restoration of a full-thickness cartilage defect treated with microfracture in a rabbit model.

**Methods:**

Full-thickness cartilage defects (3.5 mm in diameter and 3 mm in depth) were created in the patellar groove of the right femurs of 24 female New Zealand White rabbits. The rabbits were divided into two groups (12 in each group) based on postsurgery treatment differences, as follows: microfracture plus weekly intraarticular injection of KGN (group 1) and microfracture plus dimethyl sulfoxide (group 2). Six rabbits from each group were illed at 4 and 12 weeks after surgery, and their knees were harvested. The outcome was assessed both macroscopically, by using the International Cartilage Repair Society (ICRS) macroscopic evaluation system, and histologically, by using the modified O’Driscoll histologic scoring system. Immunohistochemistry for type II and I collagen was also conducted.

**Results:**

At 4 weeks, group 1 showed better defect filling and a greater number of chondrocyte-like cells compared with group 2. At 12 weeks, group 1 showed statistically significantly higher ICRS scores and modified O’Driscoll scores compared with group 2. More hyaline cartilage-like tissue was found in the defects of group 1 at 12 weeks.

**Conclusions:**

Intraarticular injection of KGN enhances the quality of full-thickness cartilage defects repair after microfracture, with better defect filling and increased hyaline-like cartilage formation.

## Introduction

Full-thickness cartilage defects commonly occur and cannot heal spontaneously because of avascular and hypocellular processes; however, even focal cartilage defects progressively lead to the degeneration of the entire joint. To restore cartilage defects, various kinds of strategies have been developed [1-4]. Microfracture is one of the most common techniques used to treat full-thickness cartilage defects because of its low cost and simple method. After the subchondral bone is perforated to initiate bleeding, mesenchymal stem cells (MSCs) are recruited into the defects and differentiated into repair tissue. Both research types using rabbit models and short-term clinical studies have demonstrated that microfracture significantly relieves pain and improves knee function [5-7].

However, many researchers have observed that the long-term results after microfracture are poor and that microfracture is limited in treating cartilage defects in elderly patients. Von Keudel *et al*. [8] conducted a long-term follow-up study to evaluate the results of microfracture by using MRI scans and showed poor outcomes after an average of 48 months after surgery. A meta-analysis conducted by Mithoefer *et al*. [5] reported that the postoperation revision rate of the microfracture technique was approximately 2.5% at less than 24 months and increased to 2% to 31% at 24 months or longer after the operation. Indeed, microfracture does not generate pure hyaline cartilage. Specifically, the main repair tissue after microfracture is fibrocartilage, which primarily contains type I collagen [9-11]. Fibrocartilage is mechanically weaker than hyaline cartilage and degenerates easily [12,13]. To generate pure hyaline cartilage without fibrous and hypertrophic tissues in cartilage defects remains challenging.

Numerous studies have been conducted to enhance the hyaline quality of repair tissue in cartilage defects after microfracture surgery. Growth factors, platelet-rich plasma, and various types of scaffolds have been used to improve the repair quality after microfracture [14-17]. Positive effects were observed in the majority of these studies; however, no strategies exist that can fully replicate the biomechanical features of hyaline cartilage because the composition and organization of the repair tissue are insufficient [18]. Furthermore, several reagents and materials that were used in previous studies may even have adverse effects; therefore, novel strategies that can improve the repair quality after microfracture are still needed.

Recently, in a high-throughput screen, Johnson *et al.* [19] discovered a small molecule, kartogenin (KGN), that directs human bone marrow-derived mesenchymal stem cells (BMSCs) into chondrocytes by mediating the CBFβ-RUNX1 signaling pathway. Two osteoarthritis (OA) models, an acute surgical model, and a collagenase VII-induced model were used in their study to evaluate the repair effect of KGN *in vivo*. In this study, cartilage matrix regeneration was observed with intraarticular KGN administration. In our previous study, we found that KGN also has the potential to differentiate human synovium-derived mesenchymal stem cells (SMSCs) into chondrocytes. However, no studies have investigated the effect of intraarticular KGN injection on cartilage restoration after microfracture. The purpose of the present study was to evaluate the quality of cartilage-defects repair after microfracture with and without intraarticular KGN injection.

## Methods

In total, 24 skeletally mature female New Zealand White rabbits weighing between 2.0 and 2.5 kg were used. All the rabbit procedures were approved by the Institutional Rabbit Care and Use Committee of Drum Tower Hospital, Medical School of Nanjing University. KGN was a kind gift from Nanjing Zhenquan Biosciences (Nanjing, China).

### Surgical procedure

**Figure 1:**
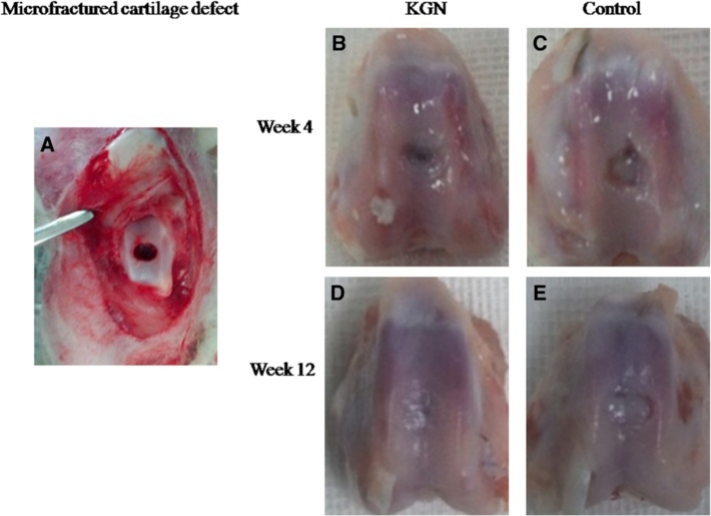
**Microfractured cartilage defect (A) and macroscopic appearance of the specimens in groups 1 (B, D) and 2 (C, E).** Gross appearance was shown at 4 (B, C) and 12 weeks (D, E). **(B)** The repair tissue covered more than 50% of the cartilage defect. **(C)** Little repair tissue was observed in the cartilage defect. **(D)** Repair tissue almost completely covered the defect in the experimental group, but the defect in the control group **(E)** was insufficiently repaired.

Each rabbit was anesthetized with an intramuscular injection of 5 mg droperidol and 0.1 g ketamine. In addition, anesthetization was maintained by slowly administering ketamine and diazepam through an auricular vein during the operation. The rabbit was placed in the supine position, and a medial parapatellar approach was used to operate on the right knee joint. Then, the patella was dislocated laterally to expose the articular surface. A full-thickness osteochondral defect (3.5 mm in diameter and 3 mm in depth) was made in the center of the trochlear groove by using an osteoarticular transplantation system. Then microfracture was performed with a 0.35 (0.9 mm) Kirschner wire (Figure 1A).

### Postoperative treatment

After surgery, the rabbits were allowed free movement in their cages; their limbs were allowed to bear the entire body weight; and their general health status was monitored by a veterinarian.

### KGN and DMSO administration

The rabbits were randomly divided into the following two groups: the experimental group (Group 1) and the control group (Group 2) (12 in each group). The KGN stock solution was prepared by directly dissolving KGN in DMSO, and the stock solution was diluted 10^5^-fold with PBS to a concentration of 10 μ*M* to prepare the working solution. In total, 0.3 ml of 10 μ*M* KGN was injected into the operated-on joints of the rabbits in Group 1 once a week for 4 or 12 weeks. The control rabbits received an intraarticular injection of DMSO diluted10^5^-fold. The injection procedure was performed by using a 21-gauge needle and a superior-lateral approach by the same operator.

### Macroscopic evaluation

The rabbits in each group were killed at 4 and 12 weeks (six rabbits at each time interval in each group), and the operated-on knees were harvested. The defect sites were photographed and blindly scored by three different investigators by using the International Cartilage Repair Society (ICRS) scoring system [20] (Table 1).

**Table 1:** **International Cartilage Repair Society macroscopic evaluation of cartilage repair**

**Categories**	**Score**
Degree of defect repair	
In level with surrounding cartilage	4
75% repair of defect depth	3
50% repair of defect depth	2
25% repair of defect depth	1
No repair of defect depth	0
Integration to border zone	
Complete integration with surrounding cartilage	4
Demarcating border /////_1 mm	3
Three-quarters of graft integrated, one-quarter with a notable border _1//// mm in width	2
One-half of graft integrated with surrounding cartilage, one-half with a notable border/// _1 mm	1
From no contact to one-quarter of graft integrated with surrounding cartilage	0
Macroscopic appearance	
Intact smooth surface	4
Fibrillated surface	3
Small, scattered fissures or cracks	2
Several small or few large fissures	1
Total degeneration of grafted area	0
Overall repair assessment	
Grade I: normal	12
Grade II: nearly normal	8-11
Grade III: abnormal	4-7
Grade IV: severely abnormal	1-3

### Histologic evaluation

After macroscopic evaluation, the specimens were fixed in 10% formalin for 7 days and decalcified in 15% ethylenediaminetetraacetate (EDTA)-buffered saline solution (Sunshine, Nanjing, China) for approximately 14 days. The specimens were dehydrated with serial ethanol, embedded in paraffin (Sigma), and cut into 5-μm sections. The sections were stained with toluidine blue (Sigma) and safranin O/Fast Green (Sigma) and viewed under a light microscope (Olympus, ////Japan). The sections were blindly scored by three different investigators by using a modified O’Driscoll histology scoring system [15,21] (Table 2).

**Table 2:** **The modified O’Driscoll histologic score**

**Characteristic**		**Grading**
I. % Hyaline cartilage	80–100	Score
	60–80	8
	40–60	6
	20–40	4
	0–20	2
II. Structural characteristics		0
A. Surface irregularity	Smooth and intact	
	Fissures	2
	Severe disruption, fibrillation	1
B. Structural integrity	Normal	0
	Slight disruption, including cysts	2
	Severe lack of integration	1
C. Thickness	100% of normal adjacent cartilage	0
	50% to 100% or thicker than normal	2
	0–50%	1
D. Bonding to adjacent cartilage	Bonded at both ends of graft	0
	Bonded at one end/partially both ends	2
	Not bonded	1
III. Freedom from cellular changes of degeneration	Normal cellularity, no clusters	0
	Slight hypocellularity, <25% chondrocyte clusters	2
	Moderate hypocellularity, >25% clusters	1
IV. Freedom from degenerate changes in adjacent cartilage	Normal cellularity, no clusters, normal staining	0
	Normal cellularity, mild clusters, moderate staining	3
	Mild or mod hypocellularity, slight staining	2
	Severe hypocellularity, slight staining	1
V. Reconstitution of subchondral bone	Complete reconstitution	0
	Greater than 50% recon	2
	50% or less recon	1
VI. Bonding of repair cartilage to *de novo* subchondral bone	Complete and uninterrupted	0
	<100% but >50% recon	2
	<50% complete	1
VII. Safrinin O staining	>80% homogeneous positive stain	0
	40%–80% homogeneous positive stain	2
	<40% homogeneous positive stain	1
	Total score	0
		Max27

### Immunohistochemistry

Regarding the immunohistochemical staining, the sections were incubated with 0.4% pepsin at 37°C for 1 hour for antigen retrieval. Endogenous peroxidase was blocked with 3% H_2_O_2_ in methanol for 30 minutes. To block nonspecific protein binding, 1% bovine serum albumin (BSA) solution (Sigma) was used. Monoclonal mouse anti-col II primary antibody (Calbiochem, Merck Millipore, ////1:100 dilution) and anti-col I primary antibody (Abcam) were used to incubate the samples at 4°C overnight. Biotinylated secondary anti-mouse antibody (GE Healthcare; 1:500 dilution) was used to incubate the sections for 1 hour. The color reaction was developed with 3′,3-diaminobenzidine (DAB) solution (Sigma), and 1% BSA solution was used as a control.

### Data analysis

All the results are expressed as the mean ± standard deviation. The differences between the groups for macroscopic and histologic scores were analyzed with the unpaired *t* test, and a 95% confidence interval was also calculated. The significance was set at *P* ≤ 0.05. All the data were analyzed by using SPSS software (version 20.0; IBM, USA).

## Results

### The health status of the rabbits

No rabbits died because of the surgical procedure or post-operative treatment, and all rabbits were able to load their limbs with no lameness. No evidence of surgery-related complications (e.g., joint or surgery site infection) was observed. No signs of adverse effects related to KGN injection were detected.

### Macroscopic evaluation

At 4 weeks, repair tissue covered more than 50% of the cartilage defects in group 1; however, relatively little fibrous tissue was observed in group 2 (Figure 1B, C). At 12 weeks, the cartilage defects in group 1 were almost 100% filled with an obscure demarcation from surrounding cartilage (Figure 1D). However, in group 2, fibrous-like repair tissue covered approximately 80% of the defects with large penetrating cracks or fissures in many specimens (Figure 1E). At 12 weeks, the mean ICRS score for group 1 was 10.0 ± 1.1 compared with a mean score of 7.8 ± 1.3 for group 2 (P < 0.02) (Table 3).

**Table 3:** **Results of macroscopic evaluation at 3 months**

	**Mean score ± SD**			**95% Confidence interval**	
**Time periods**	**Group 1**	**Group 2**	**P value**	**Lower limit**	**Upper limit**
Three months	10.0 ± 1.1	7.8 ± 1.3	0.012*	0.60	3.73

*Statistically significant deerence.

### Histological evaluation of the full-thickness cartilage defects treated with microfracture and KGN

**Figure 2:**
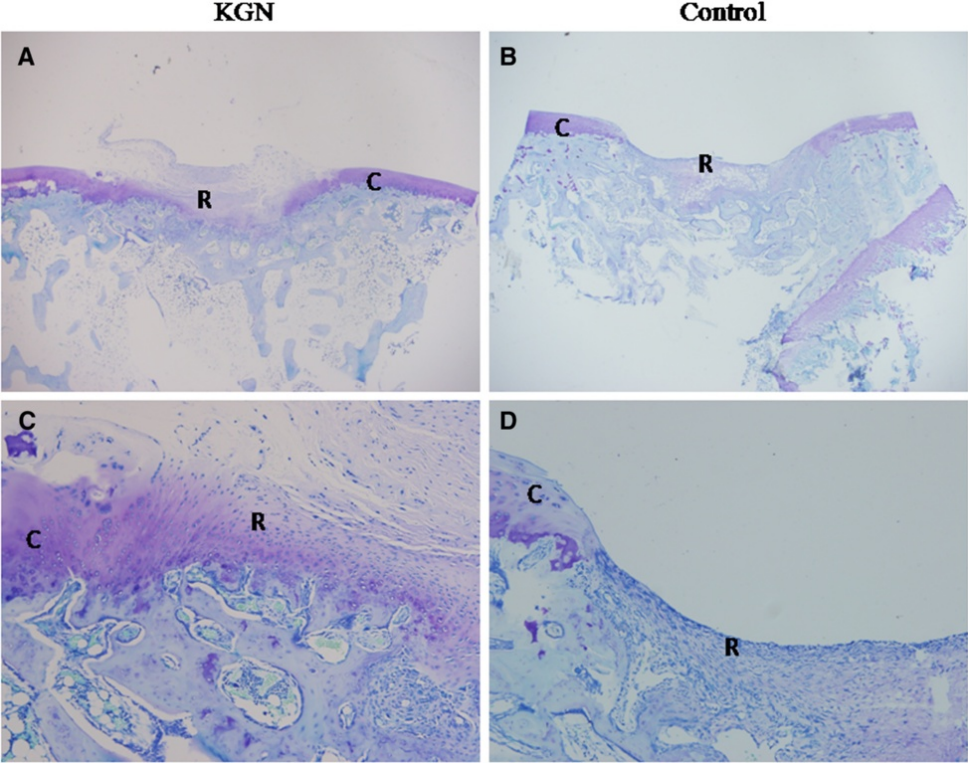
**Representative toluidine blue staining of the sections in experimental group (A, C) and control group (B, D) at 4 weeks. (A)** Slightly stained repair tissue (R) covered more than 50% of the cartilage defect, although the surface was not smooth. At higher magnification (C), Toluidine blue staining of repair tissue (R) near native cartilage (C) was intense, and the integration was good. No tidemark formation was observed. **(B)** Negatively stained repair tissue (R) covered less than 50% of the cartilage defect. At higher magnification **(D)**, only fibrous tissue was observed in the defect, and no tidemark formation was found. The left column images are magnified 20X, the right column images are magnified 100X.

**Figure 3:**
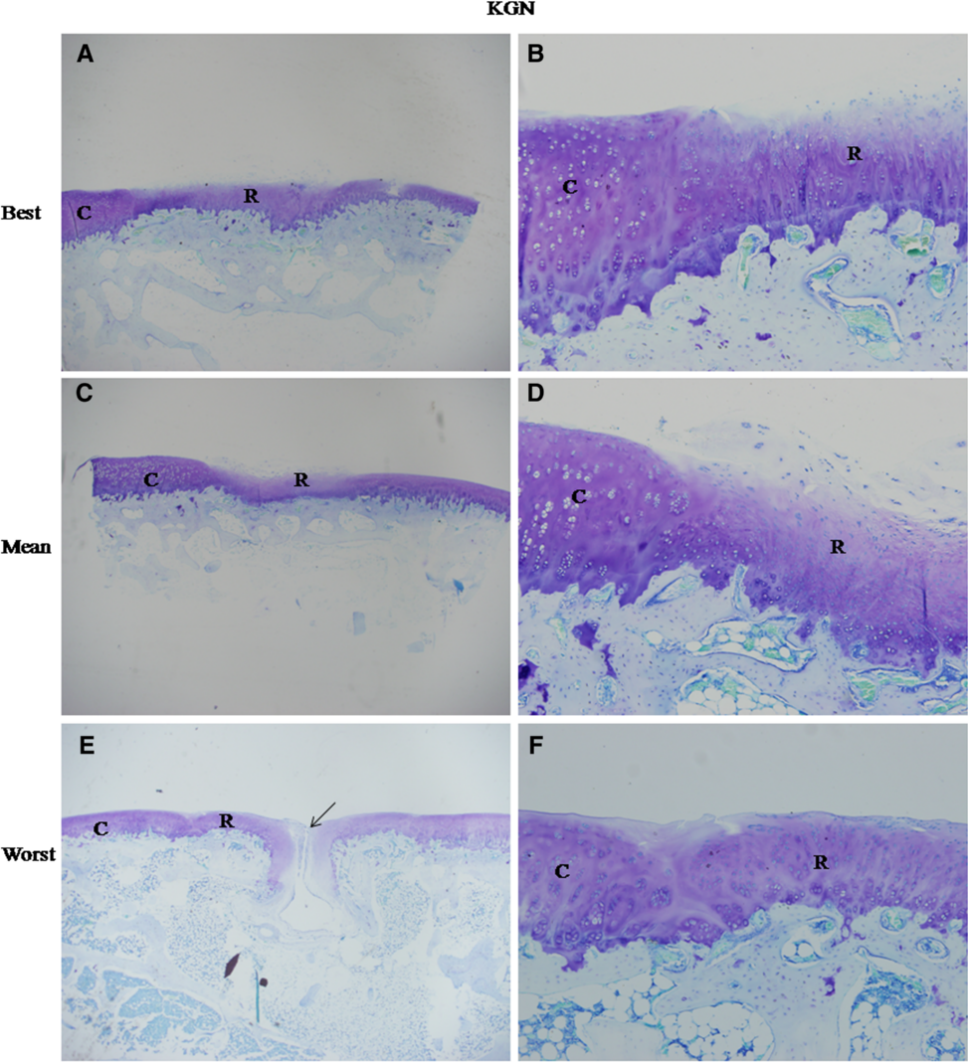
**Best, mean and worst results of toluidine blue staining in experimental group at 12 weeks. (A)** The repair tissue (R) almost completely covered the cartilage defect flush with that of native cartilage (C). The thickness of the repair tissue was similar with that of the native tissue. At higher magnification **(B)**, the demarcation between the repair tissue and the surrounding cartilage was almost undistinguishable. The repair tissue was well organized with nearly normal cell distribution. **(C)** The repair tissue (R) almost completely filled the cartilage defect. No fissures were observed. The repair tissue was thinner than adjacent healthy cartilage. At higher magnification **(D)**, No clear clefs were observed between the repair tissue and the native cartilage, although the demarcation was still distinguishable. The organization of the repair tissue was poor. **(E)** Large cracks (arrow) were seen in the center of the repair tissue (R). At higher magnification **(F)**, the integration between the repair tissue and the adjacent cartilage was good. The left column images are magnified 20X, and the right column images are magnified 100X.

**Figure 4:**
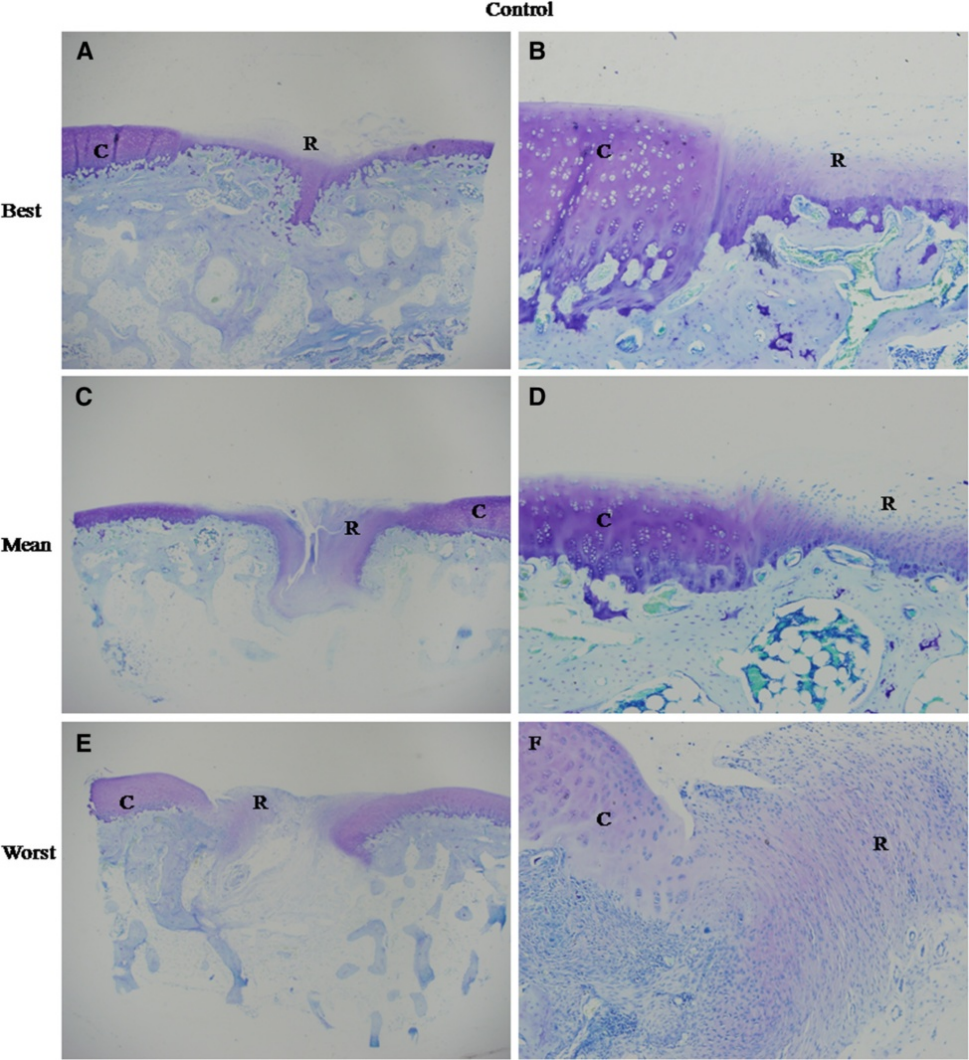
**Best, mean and worst results of safranin O staining in experimental (A, C and E) and control group (B, D and F) at 12 weeks. (A)** The repair tissue showed homogenous intense safranin O staining. Tidemark formation was observed, and subchondral bone formation was good. The surface was not smooth, with fibrous tissue existing. **(C)** The staining of the repair tissue was homogenously intense. No fissues or cracks were seen. But, no tidemark formation was observed. **(E)** Subchondral bone formation was poor with a large crack in the center of the repair tissue. But, safranin O staining was still intense in the extracellular matrix (ECM) of the repair tissue. No tidemark formation was found. **(B)** The ECM of the repair tissue was stained slightly and heterogeneously. The surface was not regular. No tidemark formation was seen. Subchondral bone formation was not good. **(D)** The repair tissue was stained negatively. No tidemark formation was seen, and subchondral bone formation was poor. **(F)** Little subchondral bone was seen. Safranin O staining was negative in the ECM of the repair tissue. All the magnification was 20X.

Histological analysis of the repair tissue in group 1 showed the formation of hyaline cartilage-like tissue. At 4 weeks, Toluidine blue staining revealed a larger amount of positively stained repair tissue in the defects in group 1 than in group 2 (Figure 2A, C). At 12 weeks, Toluidine blue staining (Figure 3) and Safranin O staining (Figure 4A, C and E) were used to assess the proteoglycans content in the repair tissue and showed three grades of intensity. Intense homogeneous staining of Toluidine blue and Safranin O at 12 weeks demonstrated that the defects in the majority of the specimens (5 of 6) were almost completely reconstructed (Figure 3; Figure 4A, C and E). Both the lateral and basal integration were satisfactory, and the subchondral bone was properly formed. In addition, well organized repair tissue was observed in the best repaired specimen (1 of 6) with nearly complete tidemark formation and nearly normal cell distribution. However, a penetrating crack was still detected in the repair tissue of the least repaired specimen (1 of 6).

### Histological evaluation of the full-thickness cartilage defects treated with microfracture and DMSO

**Figure 5:**
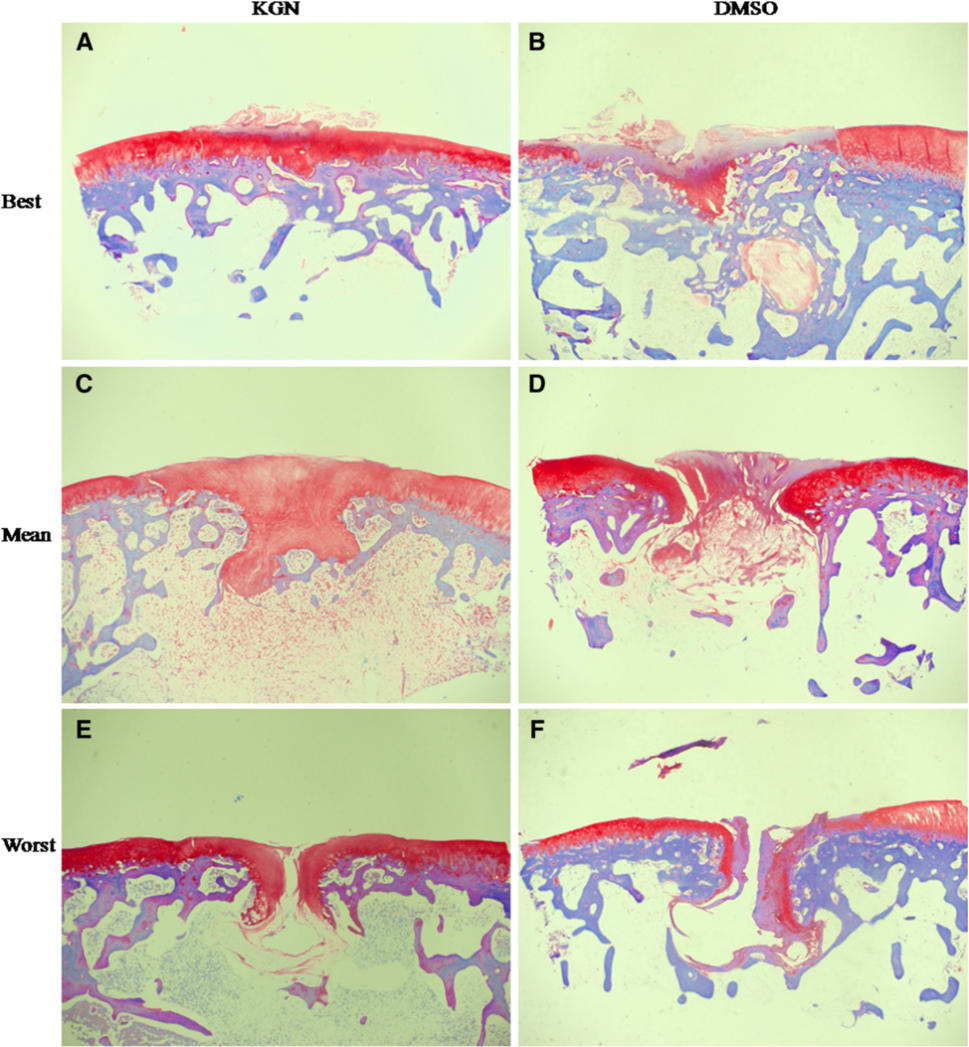
**Best, mean and worst results of toluidine blue staining in control group at 12 weeks. (A)** The cartilage defect was almost fully covered by repair tissue (R), but poor tuluidine blue staining was observed. At higher magnification **(B)**, the demarcation between the repair tissue and the surrounding cartilage (C) was clear, although no clefs were observed. The organization of the repair tissue was poor with no typical cell distribution. **(C)** Clear crack or a poorly organized repair tissue (R) was observed in the defect. Subchondral bone formation was poor. At higher magnification **(D)**, the integration was slight, and the organization of the repair tissue was poor with no typical cell distribution. **(E)** membrane-like and negatively stained repair tissue (R) covered the defect. At higher magnification **(F)**, no integration was observed. No chondrocyte-like cells were seen. The left column images are magnified 20X, and the right column images are magnified 100X.

Histological analysis of group 2 showed no hyaline cartilage-like tissue formation. At 4 weeks, Toluidine blue staining (Figure 2B, D) showed that the defects were poorly covered by only fibrous-like tissue. At 12 weeks, both Toluidine blue (Figure 5) and safranin O (Figure 4B, D and F) slightly and heterogeneously stained the tissue. The demarcation between the repair tissue and the adjacent cartilage remained clear in all the samples. Subchondral bone formation was poor in the majority of the specimens (5 of 6). The repair tissue was also poorly organized, and no tidemark formation was observed. At 12 weeks, the histological scores (Table 4) in group 1 were significantly higher than those in group 2 for all the variables except for cartilage degeneration and basal integration, which did not show any significant differences between the two groups. Group 1 showed a significantly higher overall score than group 2 (18.5 ± 4.2 VS 8.3 ± 4.6, P < 0.01).

**Table 4:** **Results of histological evaluation at 3 months**

	**Mean score ± SD**			**95% Confidence interval**	
**Variable**	**Group 1**	**Group 2**	**P value**	**Lower limit**	**Upper limit**
%Hyaline cartilage	4.7 ± 3.0	1.0 ± 1.1	0.019*	0.75	6.58
Structural characteristics	6.0 ± 0.6	3.2 ± 2.0	0.009*	0.89	4.78
Freedom from cellular changes of degeneration	1.8 ± 0.4	1.2 ± 1.0	0.156	−0.30	1.64
Freedom from degenerate changes in adjacent cartilage	1.7 ± 0.8	1.3 ± 0.5	0.419	−0.55	1.21
Reconstitution of subchondral bone	1.5 ± 0.6	0.5 ± 0.6	0.010*	0.30	1.71
Bonding of repair cartilage to de novo subchondral bone	1.5 ± 0.6	0.8 ± 0.8	0.110	−0.18	1.53
Safrinin O staining	1.3 ± 0.8	0.3 ± 0.5	0.030*	0.12	1.88
Total	18.5 ± 4.2	8.3 ± 4.6	0.003*	4.46	15.87

*Statistically significant difference.

### Immunohistochemistry

**Figure 6:**
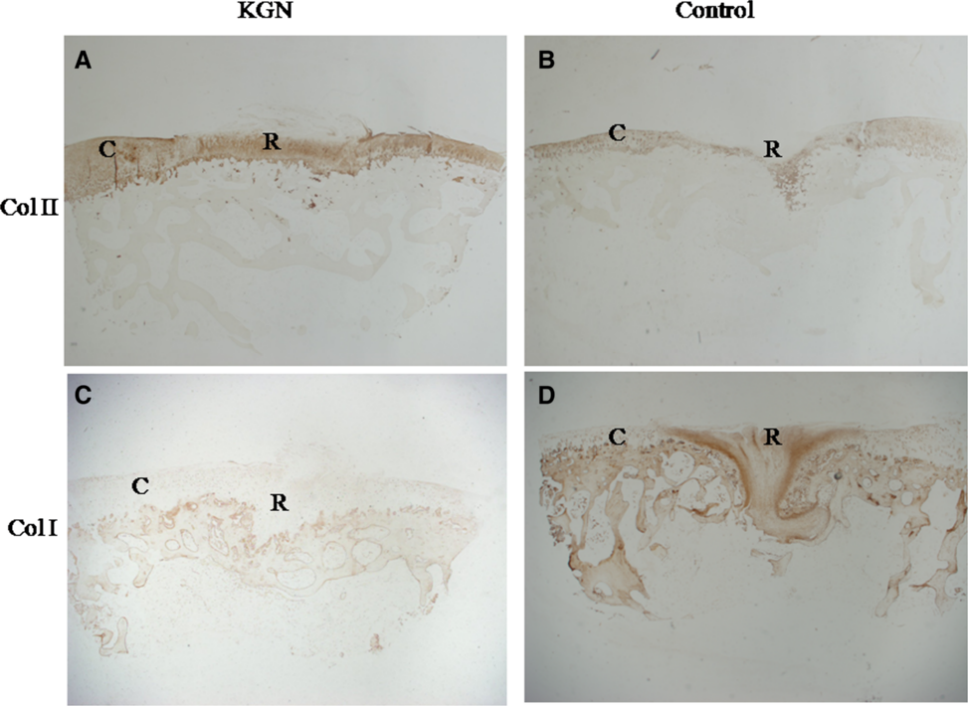
**Immunohistochemical staining of the sections in experimental (A, C) and control group (B, D) at 12 weeks.** Type I and II collagen was visualized by DAB (brown) staining. **(A)** The staining of type II collagen in repair tissue (R) was strong and similar with that of adjacent cartilage (C). **(B)** The matrix of the repair tissue (R) was stained slightly. **(C)** Almost no type I collagen staining was detected in the repair tissue (R) of group 1. **(D)** Intense type I collagen staining was observed in repair tissue (R) of group 2. All the images are magnified 20X.

In group 1, repair tissue in the majority of the samples showed strong type II collagen staining (Figure 6A) and weak type I collagen staining (Figure 6C). In group 2, most of the samples were barely stained with type II collagen (Figure 6B), and intense type I collagen staining was observed (Figure 6D).

## Discussion

This study shows that intra-articular injection of the small molecule KGN after microfracture results in significantly improved repair tissue in cartilage defects. Significant enhancements in the macroscopic ICRS and modified O’ Driscoll scores were observed in the experimental group. In addition, the repair tissue that was generated in the presence of KGN showed the characteristics of hyaline cartilage with homogeneous Safranin O staining and strong type II collagen immunoreactivity. Importantly, no adverse effects were caused by the intra-articular KGN injection.

The macroscopic observation of the specimens showed that more repair tissue covered the defects in the experimental group. We speculate that the repair tissue differentiated from the MSCs in bone marrow, synovium or adipose tissue. These MSCs may be recruited into cartilage defects after microfracture [22-24]. Subsequent studies are needed to illuminate the precise roles that each type of MSC plays in repairing these defects. Although BMSCs are no longer enriched when the subchondral bone stops bleeding [25], other sources of MSCs, such as SMSCs in joint fluid, may be recruited during the cartilage repair process [26]. In a previous study, KGN was shown to recruit MSCs in the joint fluid to cartilage damage sites [19]. The intra-articular injection of KGN in our study may enrich MSCs in cartilage defects, which may result in a higher percentage of healing in the experimental group.

In the modified O’Driscoll histology scoring system, the experimental group obtained high scores related to multiple variables, such as the content of hyaline cartilage, the structure of the repair tissue and the reconstruction of the subchondral bone. These data may have resulted from the ability of KGN to promote chondrocyte differentiation from MSCs. Repair tissue and adjacent cartilage regeneration were minimal in group 1 compared with group 2; however, no significant difference was detected. These observations support the data from two previously developed OA models [19]. We postulate that KGN also plays a role in protecting existing chondrocytes. Overall, the efficiency of intra-articular KGN administration after microfracture may be attributable to both repair effect and protective effects.

Intra-articular injection of repair-promoting agents is a simple and less invasive procedure to improve repair tissue after microfracture. Recently, many reagents such as recombinant protein-based growth factors have been used to promote cartilage repair after microfracture [15]. Growth factors have been widely believed to have a strong potency in stimulating chondrogenesis [27,28]. However, small-molecule compounds possess obvious advantages towards recombinant protein-based growth factors. First, the cost and risk of cross-species contamination are significantly lower when using small molecules [29,30]. Second, low molecular weight therapeutics is too small (<1000 KDa) to induce an immune response [31]. These properties indicate that small molecule compounds are a potential alternative to growth factors in tissue regeneration [32,33].

The KGN concentration used in our study was 10 μM because data from our previous *in vitro* study showed that 10 μM KGN displays the greatest chondrogenesis potential without cytotoxicity. Based on the consideration that KGN sustainably induces MSCs recruited from joint fluid into chondrocytes, the injection procedure was not halted until the rabbits were sacrificed. Therefore, the two duration periods of the injection procedure was 4 and 12 weeks. KGN was injected once a week to maintain an effective concentration in the knee joint; however, the precise concentration of KGN in knee joint was not monitored, which is the primary shortcoming of the present study. Future work should focus on identifying the best frequency and duration of KGN injection to promote tissue repair.

## Conclusions

In conclusion, intra-articular KGN injection enhances the quality of full-thickness cartilage defect repair after microfracture.

## Abbreviations

BMSCs: Bone marrow-derived mesenchymal stem cells
DAB: Diaminobenzidine
ECM: Extracellular matrix
EDTA: Ethylenediaminetetraacetic
KGN: Kartogenin
MSCs: Mesenchymal stem cells
OA: Osteoarthritis
SMSCs: Synovium-derived mesenchymal stem cells
